# Gut microbiota of bats: pro-mutagenic properties and possible frontiers in preventing emerging disease

**DOI:** 10.1038/s41598-021-00604-z

**Published:** 2021-10-26

**Authors:** Igor V. Popov, Maria S. Mazanko, Elizaveta D. Kulaeva, Sergey N. Golovin, Aleksey V. Malinovkin, Iraida S. Aleshukina, Anna V. Aleshukina, Evgeniya V. Prazdnova, Tatiana I. Tverdokhlebova, Michael L. Chikindas, Alexey M. Ermakov

**Affiliations:** 1 Center for Agrobiotechnology, Don State Technical University, Rostov-on-Don, Russian Federation 344000; 2 Academy of Biology and Biotechnology Named After D. I. Ivanovsky, Southern Federal University, Rostov-on-Don, Russian Federation 344090; 3 Rostov Research Institute of Microbiology and Parasitology, Rostov-on-Don, Russian Federation 344000; 4 Health Promoting Naturals Laboratory, School of Environmental and Biological Sciences, Rutgers State University, New Brunswick, NJ 08901 USA; 5 Department of General Hygiene, I.M. Sechenov First Moscow State Medical University, Moscow, Russian Federation 119991

**Keywords:** Microbiology, Bacterial physiology, Animal physiology

## Abstract

Bats are potential natural reservoirs for emerging viruses, causing deadly human diseases, such as COVID-19, MERS, SARS, Nipah, Hendra, and Ebola infections. The fundamental mechanisms by which bats are considered “living bioreactors” for emerging viruses are not fully understood. Some studies suggest that tolerance to viruses is linked to suppressing antiviral immune and inflammatory responses due to DNA damage by energy generated to fly. Our study reveals that bats' gut bacteria could also be involved in the host and its microbiota's DNA damage. We performed screening of lactic acid bacteria and bacilli isolated from bats' feces for mutagenic and oxidative activity by lux-biosensors. The pro-mutagenic activity was determined when expression of *recA* increased with the appearance of double-strand breaks in the cell DNA, while an increase of *katG* expression in the presence of hydroxyl radicals indicated antioxidant activity. We identified that most of the isolated bacteria have pro-mutagenic and antioxidant properties at the same time. This study reveals new insights into bat gut microbiota's potential involvement in antiviral response and opens new frontiers in preventing emerging diseases originating from bats.

## Introduction

Over the past years, bats have been widely studied as primary reservoirs for emerging deadly human viruses. There is direct evidence that Nipah and Hendra viruses, which lead to outbreaks in Southeast Asia and Australia, originated from bats^1–3^. The evidence of bat origin of the Ebola virus is debatable as it is based only on serological studies and detection of Ebola-related viruses in these animals^4–6^. A similar situation is with SARS-CoV, MERS-CoV, and especially SARS-CoV-2, as there is no unequivocal worldwide consensus on the origin of these viruses^7,8^. However, the involvement of bats in the interspecies transmission of emerging viruses is undeniable, as evidenced by the discovery of probable virus precursors of the abovementioned coronaviruses in them^9–11^.

Future epidemic and pandemic outbreaks of emerging viral diseases are inevitable. Even before the COVID-19, several papers were published where the possibility of occurrence of emerging zoonic bat virus has been discussed^12,13^. Therefore, one of the main priorities today is to find ways to prevent or delay the spillover of highly virulent viruses from animals to humans. Bats should be the primary target in such studies as the range of zoonotic pathogens in them is the highest among other known mammalian species^14^. Also, these animals pose an additional thread as reservoirs of emerging zoonotic infections as they are the only mammalians with the ability to fly and a relatively long lifespan^15^. This results in a high rate of contact with humans, and most importantly, other animals^16^.

The secret of viral biodiversity in bats lies in limited immune and inflammatory responses to infections. The central concept behind this unique feature is the relationship between the ability to fly and resistance to viruses mediated by high levels of free radicals produced during flight and subsequent DNA damage^17^. In addition, some suggest that the gut microbiota of bats also could be involved in tolerance to viruses. However, the exact mechanisms have not yet been revealed^18^.

In this study, we screened lactic acid bacteria and bacilli isolated from *Nyctalus noctula*, *Pipistrellus kuhlii*, and *Eptesicus serotinus* bats' feces for oxidative and mutagenic activity *in vitro* using lux-biosensors.

## Results

### Mass-spectrometry identification

**Figure 1:**
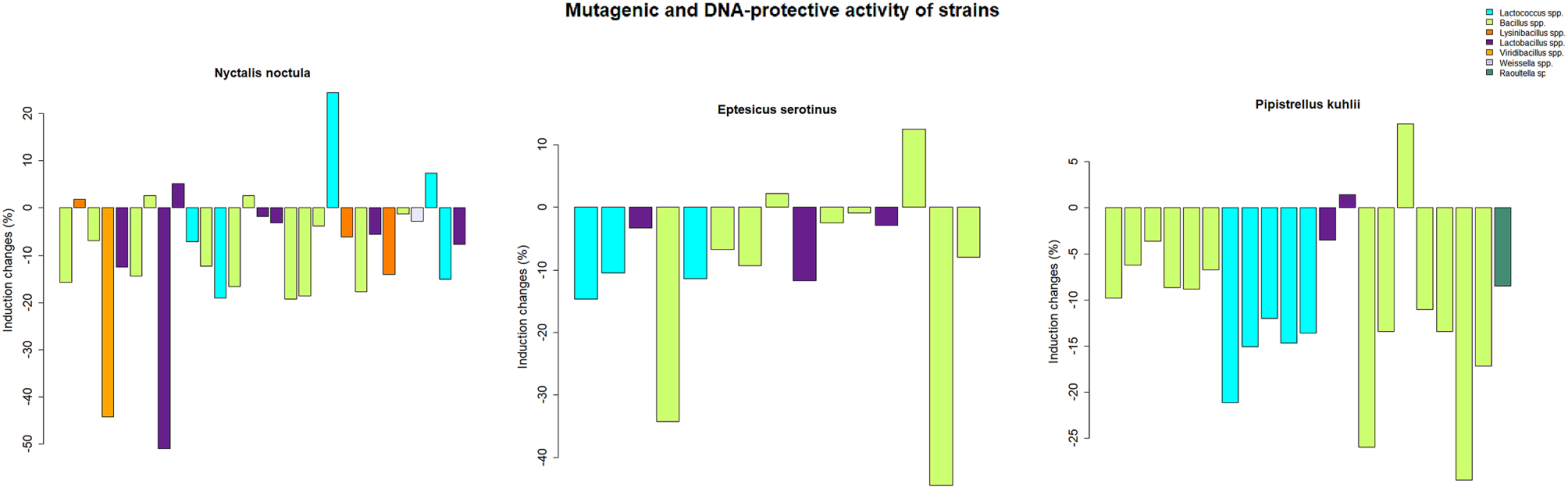
Pro-mutagenic and DNA-protective activity of bats guts lactic acid bacteria and bacilli measured with the *E. coli* RecA-biosensor. The activity was measured relative to dioxidine. The barplot legend represents groups of isolates identified by mass spectrometry.

**Figure 2:**
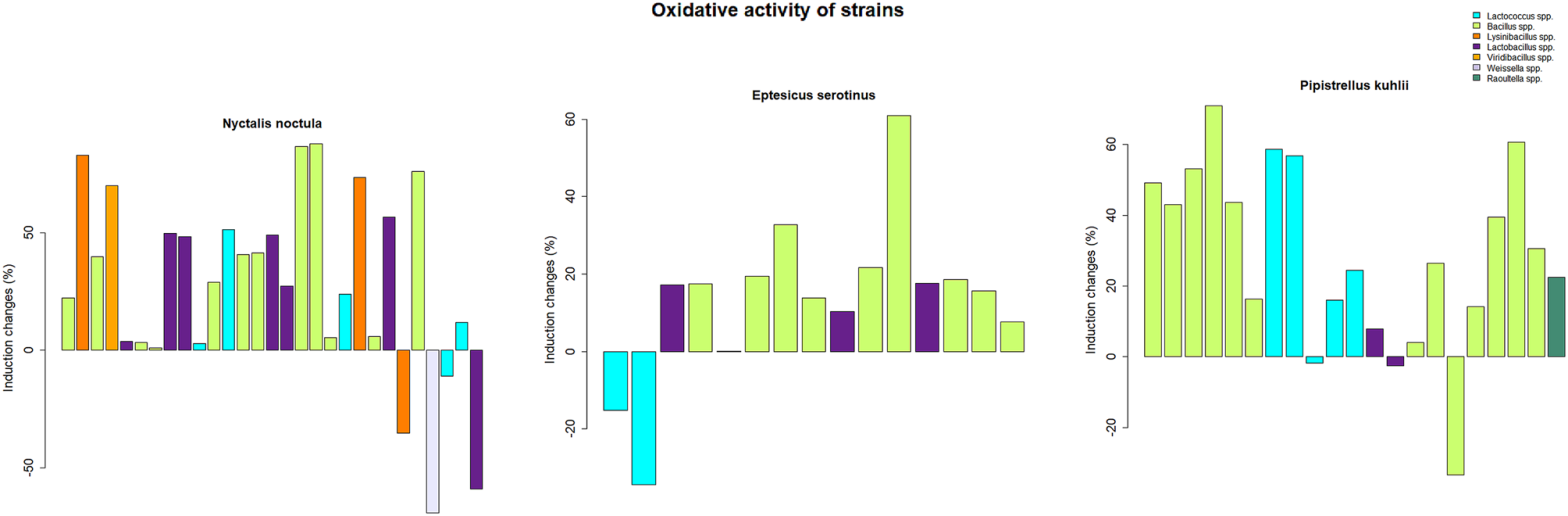
Prooxidant and antioxidant activity of bats guts lactic acid bacteria and bacilli measured with the *E. coli* Kat-biosensor. The activity was measured relative to peroxide. The barplot legend represents groups of isolates identified by mass spectrometry and isolated from individual animals.

Sixty-five isolates of lactic acid bacteria and bacilli were isolated from the litter according to the following parameters: growth on MRS agar medium with sorbic acid (pH 6.4) in a microaerophilic chamber, tinctorial properties (Gram-positive bacilli and coccobacilli), and mass spectrometric biotyping (Figs. 1, 2). Out of 89 bats, lactic acid bacteria and bacilli were isolated only from 59 animals. For the lux-biosensors study, we used microorganisms identified by mass spectrometry to a genus level. Detailed results of mass spectrometric biotyping are shown in supplementary Table 1S.

### Mutagenic and antioxidant properties of bat gut commensals

**Figure 3:**
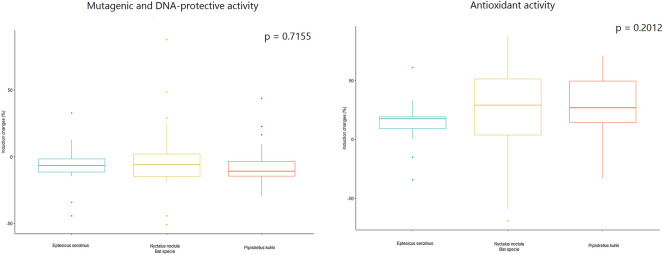
Results of the comparison of median values of mutagenic and antioxidant activity of lactobacilli and spore-forming bacilli isolates between different bat species by the Kruskal–Wallace test.

**Figure 4:**
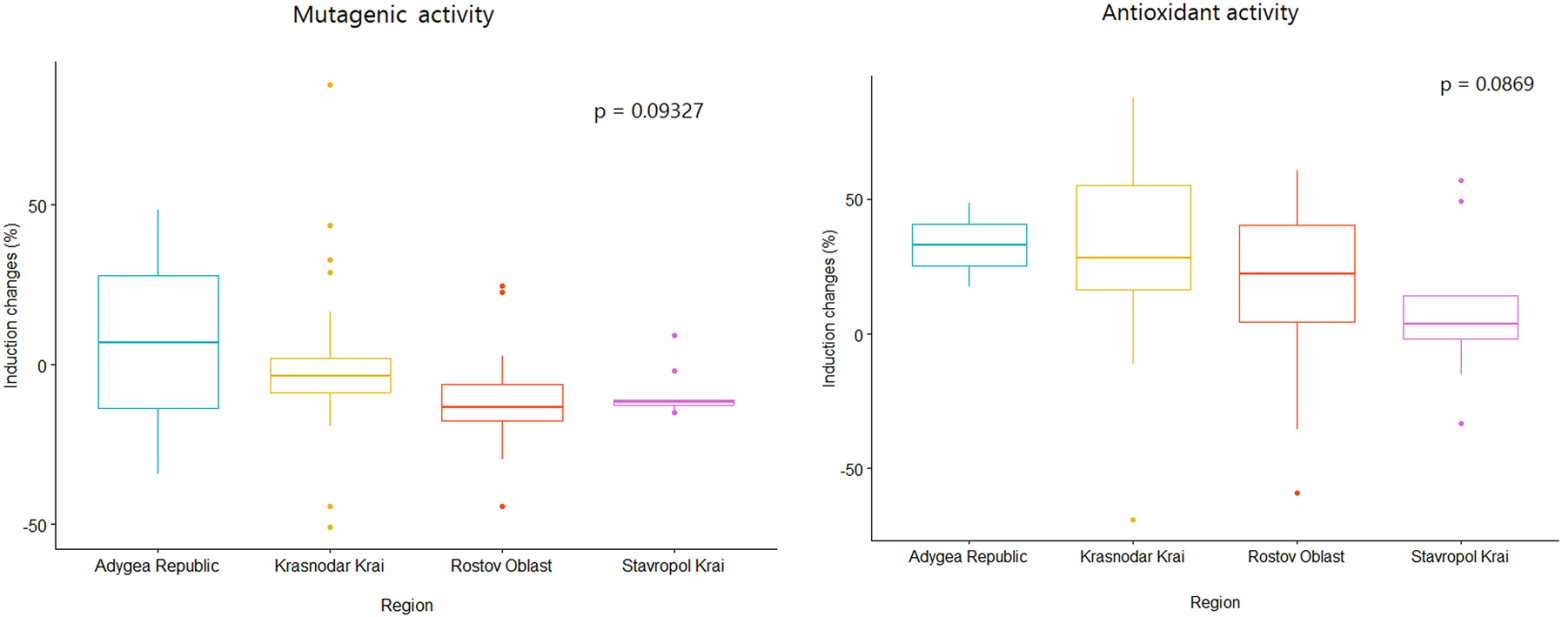
Results of the comparison of median values of mutagenic and antioxidant activity of lactobacilli and spore-forming bacilli isolates from bats of different regions by the Kruskal–Wallace test.

**Figure 5:**
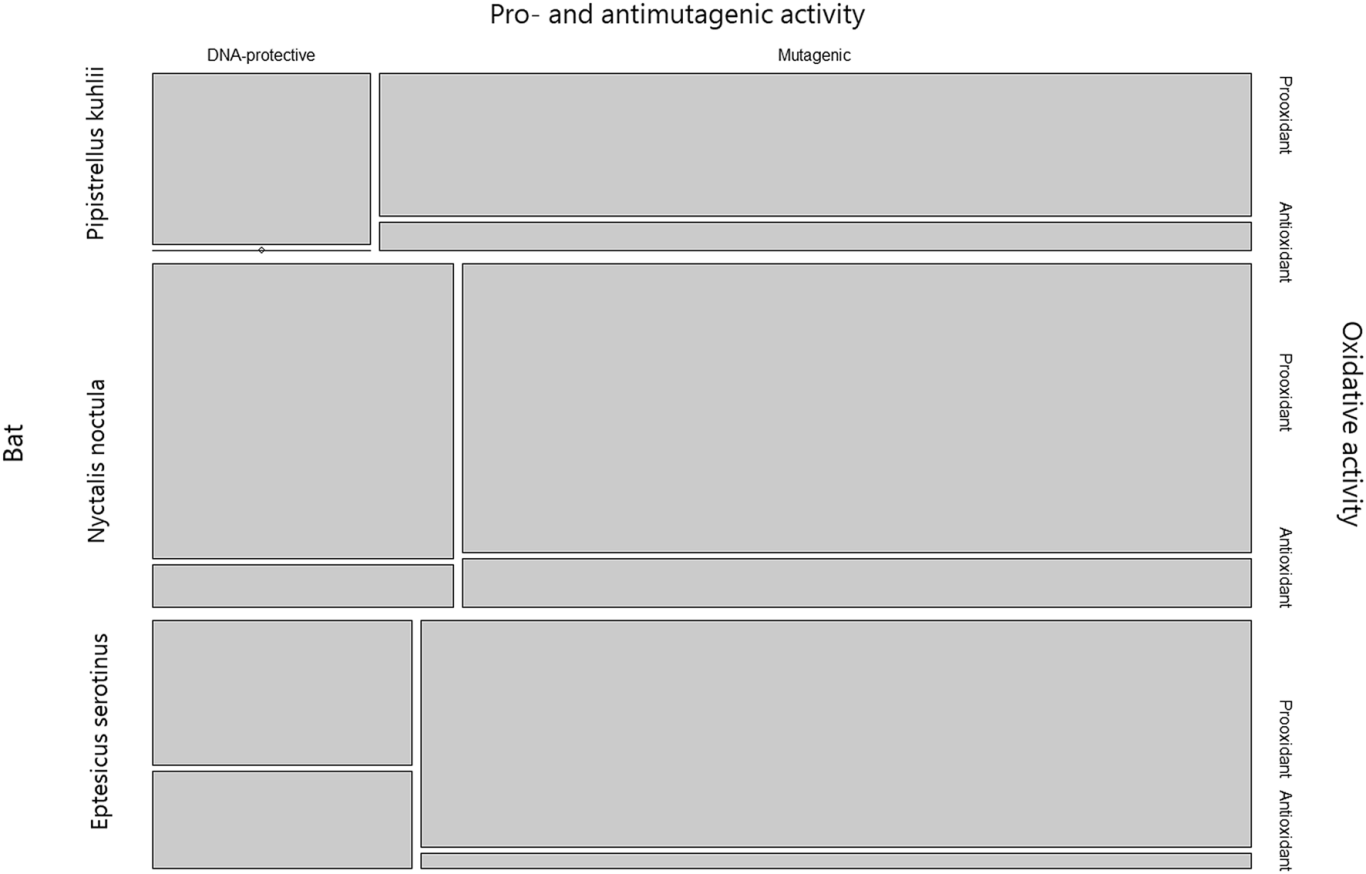
Mosaic plot representing the presence of the combination of pro-mutagenic or DNA-protective and pro- or antioxidant properties of bacteria isolated from bats feces.

According to the lux-biosensors study, most isolated lactic acid bacteria and bacilli have pro-mutagenic (Fig. 1) and antioxidant (Fig. 2) properties. Pro-mutagenic and prooxidant activities were considered when induction changes based on calculations of KatG/RecA expressions reached negative values, while DNA-protective and antioxidant properties were considered at positive values. Only 9 out of 65 isolates showed a beneficial DNA-protective effect, while most isolates except for seven showed beneficial antioxidant properties. The mean of pro-mutagenic effect evaluated and calculated relative to dioxidine is -4.796%. The mean of antioxidant effect evaluated and calculated relative to peroxide is 24.926 %. Thus, we did not observe a significant association between the mutagenic and oxidative properties (Table 1). Also, there were no statistical differences in mutagenic and oxidative activities in lactic acid bacteria and bacilli isolated from different bat species, which indicates that all isolated bacteria from bat species included in the study have similar properties: both pro-mutagenic (Fig. 3) and antioxidant (Fig. 4). According to the mosaic plot, most of the isolated bacteria have pro-mutagenic and antioxidant properties. At the same time, there is an almost complete absence of isolates with DNA-protective (anti-mutagenic) and prooxidant activity with little presence of such bacteria in *P. kuhlii and E. serotinus* (Fig. 5).

**Table 1:** Table representing results of Fisher exact test. There is no significant relationship between the pro- or antioxidant and pro-mutagenic or DNA-protective properties of isolated lactic acid bacteria and bacilli, most likely because of different mechanisms involved in providing these properties.

Bat specie	Oxidative activity	Mutagenic activity		Row Total	*p* value
		DNA-protective	Pro-mutagenic		
All species	Antioxidant	13	43	56	0.6775
	Prooxidant	3	6	9	
	Column Total	16	49	65	
*E. serotinus*	Antioxidant	3	10	13	1
	Prooxidant	0	2	2	
	Column Total	3	12	15	
*N. noctula*	Antioxidant	7	18	25	1
	Prooxidant	1	3	4	
	Column Total	8	21	65	
*P. kuhlii*	Antioxidant	3	15	18	1
	Prooxidant	2	1	3	
	Column Total	5	16	21

## Discussion

Bats are one of the most dangerous reservoirs of viral infections among mammals due to their ability to fly and spread viruses capable of interspecies spillover. The proper mechanisms making these animals perfect "living bioreactors" for emerging viruses due to viral immune tolerance and subsequent unpredictable replication and recombination of viruses are still not fully understood^16^. Luo et al. suggest that the gut microbiota of bats can be involved in the unique antiviral response of these animals, even naming it a "missing link" between the ability of flight and tolerance for viruses in bats^18^. Our study opens new frontiers in this concept, revealing expressed pro-mutagenic and antioxidant activities of bat gut bacteria at the same time.

Unlike other animals, bats' gut microbiota composition strongly depends on their environment rather than on an evolutionary predisposition to host-specific bacteria^19^. This is related to the following features of the bat gut. Intestines in bats are comparatively shorter from one-third to one-fifth than in animals of the same size due to the absence of the ascending and transverse colon^20,21^. Some bat species do not even have a whole colon^22^. Additionally, most bat species lack cecum and appendix, which are essential parts of the gastrointestinal tract for the gut microbial community in other animals^21,23^. The shorter length of the gastrointestinal tract and absence of some of its parts results in reduced transit time of food digest compared to mammalians of the same size^24^. It should be noted that along with the rapid chyme movement through the intestine, a relatively high metabolic rate has been described in bats due to energy expenditure on flights^17^. All of these features contribute to a different environment from other animals for bats gut bacteria. Most important is lower anaerobic volumes, which reduce resident anaerobic gut microbiota and increase the proportion of transient environmental microbes^25^. Our study supports this statement, as we did not isolate anaerobic or microaerophilic bacteria from the examined bats. The reason for this could be the absence or low numbers of lactic acid bacteria and bacilli in bats' feces. For studying microbial biodiversity in bats, other screening methods should be used, for example, PCR^24^. Our study is aimed at investigating mutagenic and oxidative properties of bat gut lactobacilli and spore-forming bacilli.

We used lux-biosensors to determine the properties of the microorganisms isolated from the bats' feces. Lux-biosensors are *E. coli* MG1655, containing plasmids with the operon luxCDABE of *Photorhabdus luminescens* put under the control of the *E. coli recA* and *katG* promoters. The expression of *recA* increases with the appearance of double-strand breaks in the cell DNA, which leads to activation of the SOS response system^26,27^, while the expression of *katG* increases in the presence of hydroxyl radicals^28^.

As shown in Fig. 1, the supernatants of most of the studied bacteria increased *recA* induction when supplemented simultaneously with the DNA damage inducer. However, it should be noted that the supernatants themselves did not cause a significant change in *recA* expression.

Since lux-biosensors do not show double-stranded breaks in the cell DNA, but only the cell response to them, two occasions can lead to the increase *recA* expression in the presence of metabolites. First, bacterial metabolites and the dioxidin used as an inducer can act synergistically as the number of double-strand breaks in the cell increases compared with the action of dioxidin alone. Therefore, the cellular response to damage also increases. On the other hand, metabolites can directly enhance the cell response by interfering with the SOS response in the early stages at the same level of DNA damage.

Initially, RecA protein expression is at a low level. The *recA* promoter, like other SOS response protein promoters, is repressed by the LexA protein. When double-strand breaks occur, RecA protein binds to the damaged ssDNA and forms activated RecA protein, under the influence of which LexA protein is autocatalytically cleaved, leading to a rapid increase in the expression of *recA* and other genes involved in the SOS response^26–28^. It can be speculated that metabolites of bat intestinal bacteria can increase the affinity of RecA for ssDNA or interact with LexA, accelerating its autocatalysis. The following can only be considered as a very cautious assumption; however, in eukaryotes, there is a system of homologous repair of double-strand breaks, and the RAD51 protein playing an essential role in its regulation^29^. Moreover, RAD51 protein is homologous and similar to RecA^30,31^. Thus, it is possible that the action of metabolites of the intestinal microbiota can enhance the SOS-response of bacteria and the processes occurring in the eukaryotic cells of the host. On the one hand, the homologous repair system works only in dividing cells in the S- and G2-stages of the cell cycle. So it does not belong to the primary cell repair systems^32^. On the other hand, due to the ability to fly, the cells of bats produce increased levels of prooxidants^17^, which leads to an increase in DNA damage, among other things. There are many genetically anchored adaptations in bats to protect various systems from prooxidants^17,33^, including enhanced DNA repair systems^34,35^. It is possible that the gut microbiota of bats also contributes to the enhanced DNA repair systems of bats.

RAD51 also plays a prominent role in the replication of viruses, including single-stranded RNA viruses such as coronaviruses and retroviruses. For instance, the human immunodeficiency virus can enhance RAD51 expression, increasing cell survival after treatment with genotoxic agents and the transcription level of viral proteins^36,37^. On the other hand, high levels of RAD51 limit the integration of HIV into cells^38–40^. There was a positive correlation between RAD51 expression and the proviral load of the human T-lymphotropic virus^41^. Compound B02, a human RAD51 inhibitor, showed activity against SARS-CoV-2 by inhibiting its replication^42^. The resistance of volatiles to viruses may depend, among other things, on the regulation of RAD51, in which the gut microbiota may be involved.

This study speculates on new possible mechanisms in the bat-gut bacteria relationship, particularly pro-mutagenic and antioxidant action of isolated lactic acid bacteria and bacilli from bats' feces. We suggest that antioxidant properties of bat gut microbiota could be a direct response to the high level of free radicals produced during flight, which increases their survival chances in bat intestines^17^. Interestingly, antioxidant properties are also described in lactic acid bacteria and bacilli studied by lux biosensors earlier, but at the same time, they showed anti-mutagenic (DNA-protective) properties, making them good candidates for probiotics^43,44^. However, mass screening of bat gut bacteria revealed that most of them have pro-mutagenic action with expressed antioxidant activity. One explanation may be that bat gut bacteria adapt to the intestinal environment of these animals, as they strive to occupy a niche that strongly depends on the environment because of unique features of bat intestines physiology. That is why they develop properties promoting DNA damage to take out other bacteria and antioxidant properties to counteract high levels of free radicals. Another explanation may lie in the transient environmental microbes, which are relatively easy to enter the bat gut because of lower anaerobic volumes. Thus, uncontrolled contact of entire bat populations with environmental bacteria, which may have pro-mutagenic properties, may affect the known mechanisms of interaction with DNA damage caused by the production of free radicals during flight and antiviral immune response altering viral tolerance of these animals^17^. This can result in unpredicted replication and recombination of emerging viruses, which could spillover to humans and cause epidemics.

Reported data on possible pro-mutagenic and antioxidant activity of bat gut bacteria places the fundament in further research of the relationship between bats, bacteria, and viruses interaction. These properties require more in-depth studies. In particular, screening gut microbiota of bats from distant regions, as the microbiota composition of Chiroptera is different in various environments^19^. We studied bats from regions with different climates and environments and did not find significant differences; however, more evidence should be obtained, especially in countries with unfavorable epizootic scenarios. Also, the metagenomic approach can be used for a more thorough screening of bat gut microbiota composition as it allows the identification of uncultivated microorganisms. Our study points to the necessity to reveal the genetic mechanisms of possible pro-mutagenic and antioxidant activity of bats gut commensals, which should be done on samples from animals from distant geographical areas and natural habitats. We also suggest that *in vitro* studies based on gastrointestinal model simulators can help reveal the relationship between the length and structure of the bats' intestine and the pro-mutagenic and antioxidant properties of their gut bacteria^45^, as the *in vivo* controlled studies in bats could be unethical, biologically-threatening, and challenging to conduct. However, the existing artificial gastrointestinal systems, such as TIM, SHIME, ESIN, DIDGI, and SIMGI, are not suitable for the mentioned studies as is, since they were not designed for bat gastrointestinal tract^45^. Therefore, they should be modified for these purposes, or a conceptually new bat simulating system should be designed. Bat-based *in vitro* gastrointestinal model can also reveal the direct relationship between lower gastrointestinal anaerobic volumes and lower anaerobic and microaerophilic gut bacteria rates, which is observed in our study by the relatively low rate of lactobacilli and spore-forming bacilli isolates. Most importantly, this study reveals the possible mechanism of bats-bacteria-viruses interaction that humankind can control by bacterial modulation of the bats' environment to prevent new emerging viruses spillover.

## Methods

### Bats fecal samples collection

The study involved fecal samples of *N. noctula* (n=43), *P. kuhlii* (n=22), and *E. serotinus* (n=24) from southern regions of Russia (Table 2) collected from April 15th to May 31st, 2021. Minimum 0.5 g of fecal samples were taken from each bat, then they were placed at sterile containers and transported to the laboratory at 7 °C.

**Table 2:** Table representing bat species, their areal, and number included in the study.

Bat species	Region	Number
*N. noctula*	Rostov region	12
	Adygea Republic	7
	Krasnodar Krai	21
	Stavropol Krai	3
*P. kuhlii*	Rostov region	7
	Adygea Republic	4
	Krasnodar Krai	8
	Stavropol Krai	3
*E. serotinus*	Rostov region	5
	Krasnodar Krai	15
	Stavropol Krai	4

### Microorganisms isolation and mass-spectrometry identification

Samples were aseptically removed from containers for subsequent extraction by grinding in sterile phosphate-buffered saline (pH 7.4) at a 1:10 ratio. Then the extracts were inoculated into a liquid MRS medium. Inoculates were incubated at 37 °C for 24 hours, then serial tenfold dilutions in sterile PBS (pH 7.4) were made. Each dilution was plated on MRS agar medium (6.4), and the cultures were incubated in a microaerophilic chamber at 37 °C for 24 hours. The selection of colonies for further research was carried out based on colony morphology, microscopy of smears with Gram stain, and catalase activity.

Pure overnight bacterial cultures were used for mass spectrometric biotyping. Sample preparation for the mass MALDI-TOF spectrometry was carried out by the direct deposition method. The study was performed on a Microflex LT instrument (Bruker Daltonics GmbH, Leipzig, Germany) using Biotyper (version 3.0) software (Bruker Daltonics GmbH, Leipzig, Germany).

### Determination of DNA-destructive and antioxidant properties using lux-biosensors

Sample preparation included inoculation of cultures into a test tube with 10 ml of liquid LB medium and cultivation for 48 hours for lactic acid bacteria and 24 hours for bacilli at a temperature of 37 °C^44,46^. Cell-free supernatants were obtained by centrifugation (Minispin-plus; Eppendorf, Leipzig, Germany) at 11000 g for 7 min.

Determination of mutagenic and oxidative activity was based on bacterial lux-biosensors - genetically modified strains of *E. coli* MG1655 (RecA-lux) and *E.coli* MG1655 (KatG-lux) responding to DNA damage and oxidative stress, respectively, by the expression of bioluminescence genes^46,47^. The strains contain plasmids with the operon luxCDABE of *Photorhabdus luminescens* put under the control of the corresponding *E. coli* promoters. This operon contains the luciferase genes and their regulators and provides the bioluminescence used as a reporter function in this test. As an inducer of DNA damage, we used dioxidine (2,3-Quinoxalinedimethanol,1,4-dioxide, Biosintez, Russia) at a 2.25·10^−5^ M concentration was used. For the induction of oxidative damage, we used hydrogen peroxide (Aquatest Ltd, Rostov on Don, Russia) at a concentration of 10^−3^ M. Bacteria were cultured in a liquid nutrient medium at 37 °C until the early- to mid-logarithmic phase. The overnight culture was diluted with fresh medium to a density of 0.01 to 0.1 McFarland unit (3·10^6^–3·10^7^ cells/mL). Density measurements were performed using a DEN-1B densitometer (Biosan, Riga, Latvia). The suspension was then incubated for 2 hours to the early logarithmic phase. Aliquots of this culture (90 μl each) were transferred into sterile microplate wells. Then we added 10 μl of the test cell-free supernatant preparations to each well with culture aliquot following the addition of 10 µl of each studied inducers (dioxidine and hydrogen peroxide). In control wells, we added 10 µl of deionized water and 10 µl of sterile distilled water.

After treatment, the plate with samples was placed in a luminometer and incubated at 30 °C. Bioluminescence intensity was measured every 10 min over a 110-min period.

A microplate reader FLUOstar Omega (BMG Labtech Germany) was used for luminescence measurements. All experiments were performed in three independent replicates.

To evaluate KatG and RecA expression, we calculated the induction factor (I_s_) according to the formula:

1 $$I_{S} = \, L_{e} /L_{k} - 1$$

where L_e_ and L_k_ are the luminescence intensities of samples from the experimental and control groups, respectively.

The index of mutagenic and oxidative activity (A, %) was calculated by the formula:

2 $${\text{A}} = \left( {1{-}{\text{I}}_{{\text{a}}} /{\text{I}}_{{\text{p}}} } \right)\cdot100\%$$

where I_p_ and I_a_ are the SOS-response induction factors in the presence of the cell-free supernatants preparation and the control, respectively.

The detailed calculation algorithms are provided in supplemented R statistical code (Supplementary File S1).

### Statistical analysis

Statistical analysis was performed using R v4.1.0 (R Foundation for Statistical Computing, Vienna, Austria). The sets obtained from the mass spectrometer were analyzed and processed, then bar charts characterizing the SOS response and oxidative activity of the isolates under study were constructed. Mutagenic and oxidative activities were then compared between different bat species. All data were not normally distributed according to the Shapiro–Wilk test. For the median comparison between groups, the Kruskal-Wallis test was used. Fisher exact test was used to evaluate the strength of the association between the pro- or antioxidant and pro-mutagenic or DNA-protective properties of isolated lactic acid bacteria and bacilli.

### Ethical statement

The experimental protocols of the reported study were approved by the ethics committee of the Don State Technical University, Rostov-on-Don, Russia (protocol number 67-43-4). Experimental procedures for this report did not include any *in vivo* studies. ARRIVE guidelines: not applicable^48^. The collection of fecal samples was carried out according to the Sanitary and Epidemiological Regulations SP 3.2.1288-03. Studies of lactobacilli and spore-forming bacilli were carried out according to the Sanitary and Epidemiological Regulations SP 1.3.2322-08.

## Supplementary Information

Supplementary Information 1.

Supplementary Information 2.
