# Supplementary material for: Gut microbiota of bats: pro-mutagenic properties and possible frontiers in preventing emerging disease
Source: Sci Rep. 2021 Oct 26;11:21075. doi: 10.1038/s41598-021-00604-z (PMC8548564; doi:10.1038/s41598-021-00604-z)
Supplement: Supplementary file 1 — Supplementary Information 1. [file 41598_2021_604_MOESM1_ESM.pdf]

```
#SOS-response activity
setx<-as.data.frame(setx)
time_induction<-as.data.frame(setx$Time)
mydata <- as.data.frame(setx$Time)
for (i in 2:9){
  mydata[,i] <-(setx[,i+8]/setx[,i])-1
}
time_protective<-as.data.frame(setx$Time)
for (i in 2:8){
  time_protective[,i]<-(1-(mydata[,i]/mydata[,9]))*100
}
colnames(time_protective)<-c("Time", "3 PB-7", "30 PB-15",      "17 НК-6", "39 PB-52",        "59 НК-47", "34 ПК-4", "22 ПК-10")

y<-time_protective[12,]
y<-y[,-1]
y<-t(t(y))
barplot(y)

#categorization
all<-cbind(y:n)
all<-t(all)
species<-as.data.frame(species)
d <- all
names <- rownames(d)
rownames(d) <- NULL
data <- cbind(names,d)
colnames(data)<-c("Number", "Rec")
mut<-merge.data.frame(data, species, by = "Number")

#barplot
mut$Rec<-as.numeric(mut$Rec)
```

```
mut$Num_Bac<-as.numeric(mut$Num_Bac)

leg= c("Lactococcus spp.", "Bacillus spp.", "Lysinibacillus spp.", "Lactobacillus spp.", "Viridibacillus spp.", "Weissella spp.", "Raoultella spp.")

barplot(height=mut$Rec, main = "Prooxidant and antioxidant activity of strains", col=ifelse(mut$Num_Bac== 0,"red",
    ifelse(mut$Num_Bac==1,"blue",
    ifelse(mut$Num_Bac==2,"black",
    ifelse(mut$Num_Bac==3, "violet",ifelse(mut$Num_Bac==4, "brown",
    ifelse(mut$Num_Bac==5, "orange",
    ifelse(mut$Num_Bac==6, "tan",

ifelse(mut$Num_Bac==7, "cyan", "white")))))))), legend = leg)

#oxidative activity

setx_1<-as.data.frame(setx_1)

time_induction<-as.data.frame(setx_1$Time)

mydata <- as.data.frame(setx_1$Time)

for (i in 2:9){

  mydata[,i] <-(setx_1[,i+8]/setx_1[,i])-1

}

time_protective<-as.data.frame(setx_1$Time)

for (i in 2:8){

  time_protective[,i]<-(1-(mydata[,i]/mydata[,9]))*100

}

colnames(time_protective)<-c("Time", "3 РВ-7", "30 РВ-15",    "17 НК-6", "39 РВ-52",    "59 НК-47", "34 ПК-4", "22 ПК-10")


y_1<-time_protective[12,]

y_1<-y_1[,-1]

y_1<-t(t(y_1))

barplot(y_1)
```

```
#categorization


all2<-cbind(y_1:n_1)

all2<-t(all2)

species<-as.data.frame(species)

d2 <- all2

names2 <- rownames(d2)

rownames(d2) <- NULL

data2 <- cbind(names2,d2)

colnames(data2)<-c("Number", "Kat")

mut<-merge.data.frame(data2, species, by = "Number")

all_mut<-merge.data.frame(mut, data, by= "Number")


#barplot

mut$Kat<-as.numeric(mut$Kat)

mut$Num_Bac<-as.numeric(mut$Num_Bac)

barplot(height=mut$Kat, main = "Prooxidant and antioxidant activity of strains",
col=ifelse(all_mut$Num_Bac== 0,"red",

                                                      ifelse(all_mut$Num_Bac==1,"blue",

                                                          ifelse(all_mut$Num_Bac==2,"black",

                                                              ifelse(all_mut$Num_Bac==3, "violet",

                                                                  ifelse(all_mut$Num_Bac==4,
"brown",

                                                                      ifelse(all_mut$Num_Bac==5,
"orange",


ifelse(all_mut$Num_Bac==6, "tan",


ifelse(all_mut$Num_Bac==7, "cyan", "white")))))))))


ES<-subset(all_mut, Bat== "Eptesicus serotinus")

NN<-subset(all_mut, Bat== "Nyctalus noctula")

PK<-subset(all_mut, Bat== "Pipistrellus kuhlii")
```

```
ES$Kat<-as.numeric(ES$Kat)
ES$Rec<-as.numeric(ES$Rec)
NN$Kat<-as.numeric(NN$Kat)
NN$Rec<-as.numeric(NN$Rec)
PK$Kat<-as.numeric(PK$Kat)
PK$Rec<-as.numeric(PK$Rec)
shapiro.test(ES$Kat)
shapiro.test(ES$Rec)
shapiro.test(NN$Kat)
shapiro.test(NN$Rec)
shapiro.test(PK$Kat)
shapiro.test(PK$Rec)
stats_Kat<-cbind(ES$Kat, NN$Kat, PK$Kat)
stats_Kat<-as.data.frame(stats_Kat)
colnames(stats_Kat)<-c("Eptesicus serotinus", "	Nyctalus noctula", "Pipistrellus kuhlii")
stats_Rec<-cbind(ES$Rec, NN$Rec, PK$Rec)
stats_Rec<-as.data.frame(stats_Rec)
colnames(stats_Rec)<-c("Eptesicus serotinus", "	Nyctalus noctula", "Pipistrellus kuhlii")
mut.num<-as.data.frame(cbind(all_mut$Bat, all_mut$Kat, all_mut$Rec))
colnames(mut.num)<-c("Bat", "Kat", "Rec")
mut.num$Kat<-as.numeric(mut.num$Kat)
mut.num$Rec<-as.numeric(mut.num$Rec)
#KRUSKALL-WALLIS
install.packages("dplyr")
library(dplyr)
cat_group<-group_by(mut.num, Bat) %>%
  summarise(
    count = n(),
    mean = mean(Kat, na.rm = TRUE),
    sd = sd(Kat, na.rm = TRUE),
    median = median(Kat, na.rm = TRUE),
    IQR = IQR(Kat, na.rm = TRUE)
```

```
  )
rec_group<-group_by(mut.num, Bat) %>%
  summarise(
    count = n(),
    mean = mean(Rec, na.rm = TRUE),
    sd = sd(Rec, na.rm = TRUE),
    median = median(Rec, na.rm = TRUE),
    IQR = IQR(Rec, na.rm = TRUE)
  )
if(!require(devtools)) install.packages("devtools")
devtools::install_github("kassambara/ggpubr")
library("ggpubr")
Kat_1<-mut.num[,-3]
Rec_1<-mut.num[,-2]
Kat_1$Bat <- ordered(Kat_1$Bat,
                    levels = c("Eptesicus serotinus", "Nyctalus noctula", "Pipistrellus kuhlii"))
Kat_1$Kat<-as.numeric(Kat_1$Kat)
ggboxplot(Kat_1, x= "Bat", y= "Kat",
          color = "Bat", palette = c("#00AFBB", "#E7B800", "#FC4E07"),
          order = c("Eptesicus serotinus", "Nyctalus noctula", "Pipistrellus kuhlii"),
          ylab = "Antioxidant activity", xlab = "Bat specie", main = "Antioxidant activity")

Rec_1$Bat <- ordered(Rec_1$Bat,
                    levels = c("Eptesicus serotinus", "Nyctalus noctula", "Pipistrellus kuhlii"))
Rec_1$Rec<-as.numeric(Rec_1$Rec)
ggboxplot(Rec_1, x= "Bat", y= "Rec",
          color = "Bat", palette = c("#00AFBB", "#E7B800", "#FC4E07"),
          order = c("Eptesicus serotinus", "Nyctalus noctula", "Pipistrellus kuhlii"),
          ylab = "Mutagenic activity", xlab = "Bat specie")
Kat.stat<-kruskal.test(Kat ~ Bat, data = Kat_1)
Rec.stat<-kruskal.test(Rec ~ Bat, data = Rec_1)
```

```
region_mut<-cbind(all_mut$Kat, all_mut$Rec, all_mut$Region)
region_mut<-as.data.frame(region_mut)
colnames(region_mut)<-c("Kat", "Rec", "Region")
region_mut$Region <- ordered(region_mut$Region,
                         levels = c("Adygea Republic", "Krasnodar Krai", "Rostov Oblast", "Stavropol Krai"))
region_mut$Rec<-as.numeric(region_mut$Rec)
ggboxplot(region_mut, x= "Region", y= "Rec",
     color = "Region", palette = c("#00AFBB", "#E7B800", "#FC4E07", "#DA70D6"),
     order = c("Adygea Republic", "Krasnodar Krai", "Rostov Oblast", "Stavropol Krai"),
     ylab = "Induction changes (%)", xlab = "Region", main = "Mutagenic and DNA-protective
activity",
     cex.lab=8)
kruskal.test(Rec ~ Region, data = region_mut)

region_mut$Kat<-as.numeric(region_mut$Kat)
ggboxplot(region_mut, x= "Region", y= "Kat",
     color = "Region", palette = c("#00AFBB", "#E7B800", "#FC4E07", "#DA70D6"),
     order = c("Adygea Republic", "Krasnodar Krai", "Rostov Oblast", "Stavropol Krai"),
     ylab = "Induction changes (%)", xlab = "Region", main = "Oxidative activity",
     cex.lab=8)
kruskal.test(Kat ~ Region, data = region_mut)

#mosaic plot

library(ggplot2)
library(vcd)
all_mut_2<-all_mut
colnames(all_mut_2)<-c("Number", "Oxidative activity", "Bat_number", "Bat", "Bacteria", "Num",
"SOS-response")
all_mut_2$`SOS-response`<- ifelse(all_mut_2$`SOS-response`<0, 0, 1)
all_mut_2$`Oxidative activity`<- ifelse(all_mut_2$`Oxidative activity`<0, 0, 1)
all_mut_2 <- na.omit(all_mut_2)
```

```
glimpse(all_mut_2)

all_mut_2$`Oxidative activity`<-as.numeric(all_mut_2$`Oxidative activity`)

mosaic(~ all_mut_2$`SOS-response` + all_mut$ | Bat, data=all_mut_2)


#Fisher exact test

ES2<-subset(all_mut_2, Bat == "ES")

ES2<-as.data.frame(cbind(ES2$Kat,ES2$Rec))

ES_table<-table(ES2)

fisher.test(ES_table)


NN2<-subset(all_mut_2, Bat == "NN")

NN2<-as.data.frame(cbind(NN2$Kat,NN2$Rec))

NN_table<-table(NN2)

fisher.test(NN_table)


PK2<-subset(all_mut_2, Bat == "PK")

PK2<-as.data.frame(cbind(PK2$Kat,PK2$Rec))

PK_table<-table(PK2)

fisher.test(PK_table)
```
