# Supplementary material for: Gut microbiota of bats: pro-mutagenic properties and possible frontiers in preventing emerging disease
Source: Sci Rep. 2021 Oct 26;11:21075. doi: 10.1038/s41598-021-00604-z (PMC8548564; doi:10.1038/s41598-021-00604-z)
Supplement: Supplementary file 2 — Supplementary Information 2. [file 41598_2021_604_MOESM2_ESM.pdf]

Animal code	Bat specie	Isolate code	Results of mass-spectrometry biotyping
2PB	Nyctalus noctula, ♀	18-2PB	*Bacillus shackletonii*
3PB	Nyctalus noctula, ♀	7-3PB	*Lactobacillus gasseri*
12PB	Nyctalus noctula, ♂	3-12 PB	*Bacillus thuringiensis*
		4-12PB	*Lysinibacillus sphaericus*
15PB	Nyctalus noctula, ♀	8-15PB	*Viridibacillus neidei*
		13-15PB	*Bacillus odysseyi*
16HK	Pipistrellus kuhlii, ♀	21-16HK	*Bacillus luciferensis*
		23-16HK	*Bacillus vallismortis*
		25-16HK	*Bacillus odysseyi*
17HK	Pipistrellus kuhlii, ♀	5-17HK	*Bacillus vallismortis*
		6-17HK	*Bacillus odysseyi*
22ПК	Eptesicus serotinus, ♀	10-22ПК	*Bacillus subtilis*
29PB	Nyctalus noctula, ♀	14-29PB	*Bacillus shackletonii*
		19-29PB	*Lactobacillus amylovorus*
30PB	Nyctalus noctula, ♂	15-30PB	*Bacillus luciferensis*
32ПК	Eptesicus serotinus, ♀	1-32ПК	*Bacillus* spp.
34ПК	Eptesicus serotinus, ♀	4-34ПК	*Bacillus* spp.
38PB	Nyctalus noctula, ♂	45-38PB	*Lactobacillus gasseri*
39PB	Nyctalus noctula, ♀	49-39PB	*Bacillus subtilis*
		52-32PB	*Bacillus subtilis*
		53-39PB	*Bacillus* spp.

40РВ	Nyctalus noctula, ♀	10-40РВ	*Lysinibacillus* spp.
41НК	Pipistrellus kuhlii, ♀	15-41НК	*Lactococcus garvieae*
		16-41НК	*Lactococcus garvieae*
42НК	Pipistrellus kuhlii, ♀	7-42НК	*Lactobacillus crispatus*
		12-42НК	*Lactobacillus crispatus*
45ПК	Eptesicus serotinus, ♀	28-45ПК	*Lactobacillus plantarum*
48ПК	Eptesicus serotinus, ♂	51-48ПК	*Bacillus mojavensis*
49РВ	Nyctalus noctula, ♀	50-49РВ	*Bacillus mojavensis*
50РВ	Nyctalus noctula, ♀	19-50РВ	*Weissella confusa*
51РВ	Nyctalus noctula, ♀	5-51РВ	*Lactobacillus* spp.
		9-51РВ	*Lactococcus garvieae*
53ПК	Eptesicus serotinus, ♀	2-53ПК	*Lactobacillus malefermentans*
		48-53ПК	*Bacillus subtilis*
57НК	Pipistrellus kuhlii, ♂	32-57НК	*Bacillus odysseyi*
58НК	Pipistrellus kuhlii, ♀	33-58НК	*Bacillus* spp.
59НК	Pipistrellus kuhlii, ♂	35-59НК	*Bacillus* spp.
		47-59НК	*Raoultella ornithinolytica*
61РВ	Nyctalus noctula, ♀	23-61РВ	*Lactobacillus satsumensis*
62ПК	Eptesicus serotinus, ♂	34-62ПК	*Bacillus odysseyi*
		36-62ПК	*Bacillus mycoides*

65РВ	Nyctalus noctula, ♂	41	*Bacillus* spp.
66РВ	Nyctalus noctula, ♂	36	*Bacillus* spp.
67РВ	Nyctalus noctula, ♀	34	*Bacillus* spp.
68РВ	Nyctalus noctula, ♀	40	*Lactococcus garvieae*
69ПК	Eptesicus serotinus, ♀	13	*Lactococcus garvieae*
70ПК	Eptesicus serotinus, ♂	39	*Bacillus odysseyi*
71НК	Pipistrellus kuhlii, ♂	49	*Bacillus* spp.
73НК	Pipistrellus kuhlii, ♀	50	*Bacillus atrophaeus*
		29	*Lactococcus garvieae*
75РВ	Nyctalus noctula, ♀	43	*Lactobacillus gasseri*
76РВ	Nyctalus noctula, ♀	27	*Bacillus thuringiensis*
77РВ	Nyctalus noctula, ♂	30	*Lactococcus garvieae*
		31	*Lactococcus garvieae*
		47	*Lysinibacillus sphaericus*
78РВ	Nyctalus noctula, ♀	48	*Bacillus mojavensis*
80ПК	Eptesicus serotinus, ♀	52	*Sporolactobacillus laevolacticus*
81ПК	Eptesicus serotinus, ♀	44	*Lactobacillus agilis*
82НК	Pipistrellus kuhlii, ♀	3	*Lactococcus garvieae*
		4	*Lactococcus garvieae*
84НК	Pipistrellus kuhlii, ♂	51	*Bacillus atrophaeus*
87РВ	Nyctalus noctula, ♀	18	*Lactobacillus coryneformis*

		37	*Lactobacillus mali*
88ПК	Eptesicus serotinus, ♀	20	*Lactococcus garvieae*
89ПК	Eptesicus serotinus, ♀	11	*Lactococcus garvieae*

Supplementary table 1S. Table representing results of mass spectrometric biotyping of microorganisms isolated from bats feces.
